# Prognosis Prediction in COVID-19 Patients through Deep Feature Space Reasoning

**DOI:** 10.3390/diagnostics13081387

**Published:** 2023-04-11

**Authors:** Jamil Ahmad, Abdul Khader Jilani Saudagar, Khalid Mahmood Malik, Muhammad Badruddin Khan, Abdullah AlTameem, Mohammed Alkhathami, Mozaherul Hoque Abul Hasanat

**Affiliations:** 1 Department of Computer Science, Islamia College Peshawar, Peshawar 25120, Pakistan; 2 Information Systems Department, Imam Mohammad Ibn Saud Islamic University (IMSIU), Riyadh 11432, Saudi Arabia; 3 Department of Computer Science and Engineering, Oakland University, Rochester, MI 48309, USA

**Keywords:** COVID-19, prognosis prediction, patient demographics, case retrieval, deep feature space reasoning

## Abstract

The COVID-19 pandemic has presented a unique challenge for physicians worldwide, as they grapple with limited data and uncertainty in diagnosing and predicting disease outcomes. In such dire circumstances, the need for innovative methods that can aid in making informed decisions with limited data is more critical than ever before. To allow prediction with limited COVID-19 data as a case study, we present a complete framework for progression and prognosis prediction in chest X-rays (CXR) through reasoning in a COVID-specific deep feature space. The proposed approach relies on a pre-trained deep learning model that has been fine-tuned specifically for COVID-19 CXRs to identify infection-sensitive features from chest radiographs. Using a neuronal attention-based mechanism, the proposed method determines dominant neural activations that lead to a feature subspace where neurons are more sensitive to COVID-related abnormalities. This process allows the input CXRs to be projected into a high-dimensional feature space where age and clinical attributes like comorbidities are associated with each CXR. The proposed method can accurately retrieve relevant cases from electronic health records (EHRs) using visual similarity, age group, and comorbidity similarities. These cases are then analyzed to gather evidence for reasoning, including diagnosis and treatment. By using a two-stage reasoning process based on the Dempster–Shafer theory of evidence, the proposed method can accurately predict the severity, progression, and prognosis of a COVID-19 patient when sufficient evidence is available. Experimental results on two large datasets show that the proposed method achieves 88% precision, 79% recall, and 83.7% F-score on the test sets.

## 1. Introduction

Image retrieval refers to the finding of visual and semantically similar images in a large collection when a user provides an input query image. Content-based medical image retrieval (CBMIR) allows retrieval of relevant medical images which contain similar disease pathologies [1,2,3,4]. For instance, in the case of COVID-19 chest X-rays (CXR), the retrieval system can be used to find images of patients having the same pathologies as the input CXR. The retrieved images can then be used to facilitate case-based reasoning and the discovery of useful features in the data. Though, reverse transcriptase-polymerase chain reaction (RT-PCR) is usually regarded as the gold standard for COVID-19 detection, radiographic examination can allow detection at early stages and helps us monitor and prevent the spread of infection, thereby leading to better patient outcomes.

Deep learning for medical image analysis has exhibited substantial progress in radiology, dermatology, ophthalmology, and pathology. Large amounts of medical imaging datasets have enabled deep learning methods to automatically learn patterns in the data, which can be useful for diagnosis and prediction. For COVID-19 detection, both CXR and computed tomography (CT) images have proven to be equally viable for detection at early stages. Though CT represents 3D volumetric data, CXRs provide many advantages, such as quick triaging, availability, accessibility, and portability. In recent years, CXRs have been used extensively by researchers to develop deep-learning methods for COVID-19 detection [5], progression detection [6], severity estimation [7], and prognosis prediction [8]. Most studies focus on training end-to-end deep learning models to predict COVID-19 progression or outcomes from CXRs [9,10,11]. However, those methods typically require large amounts of data to effectively learn essential patterns which usually are not available during the early days of a pandemic. In such situations, case-based reasoning becomes feasible where physicians analyze previous cases to diagnose or determine the course of treatment for individual patients [4]. Image retrieval methods can effectively serve to find relevant cases from a large collection of CXRs through visual analysis of image contents. The retrieved images, along with relevant clinical records of patients, can be used in a reasoning process to predict disease course and plan treatment.

This research addresses the problem of COVID-19 progression detection and prognosis prediction by utilizing relevant cases and electronic health records. A deep feature space using a fine-tuned CNN is constructed where each CXR is represented as a point in that high-dimensional space. Each point is associated with several clinical attributes which are used to determine relevance with neighboring cases. An efficient case retrieval method is proposed to find relevant cases from the archive. The reasoning module then uses those cases as evidence to perform prediction. Major contributions to this work include:

1. A COVID-specific deep features space construction and features extraction method is proposed.
2. A hybrid representation method is proposed where each patient is represented in the deep feature space using visual features encoding and the age and comorbidities are associated with every patient as ordinary variables.
3. A multi-stage case retrieval method is developed to locate relevant cases of COVID-19 patients based on CXR and clinical records.
4. A deep feature space reasoning method based on Dempster–Shafer theory is developed, which combines evidence to determine disease progression as well as predict prognosis using relevant past cases involving clinical variables and CXRs.

The rest of the manuscript is organized into various sections, including Section 2, which summarizes relevant literature addressing the issues of severity estimation, progression detection, and prognosis prediction in COVID-19 patients. The proposed method is presented in detail in Section 3. Experimental results and discussion are provided in Section 4 and the manuscript concludes with future research directions in Section 5.

## 2. Related Work

Since the COVID-19 pandemic in late 2019 and early 2020, it has been found that chest radiography imaging can be effectively used to observe and summarize lung abnormalities, including ground glass opacities and their distribution in both lungs. Extensive studies on CXR imaging have shown their potential for severity assessment on the basis of lung involvement in the infection, disease progression detection [6], and prognosis prediction. However, in the case of COVID-19, the novelty of the disease makes it more challenging even for the expert radiologists to confidently interpret the findings, particularly on CXRs [12,13,14]. Therefore, an AI-based system that learns from expert radiologists and provides consistent results could be highly valuable in such situations. In recent years, several diagnosis tools have been developed for accurate detection of COVID-19 infection. For instance, Maghdid et al. [15] and Bukhari et al. [16] evaluated AlexNet and ResNet-50 architectures, respectively, to detect COVID-19 infection with high accuracies. In a similar work, Wang et al. [17] built a custom CNN to detect COVID-19 infection in CXR images. Rajaraman et al. [18] used an iteratively pruned model ensemble for detecting COVID-19 infection in scans. The authors investigated the performance of various deep CNNs on a wide range of datasets and concluded that InceptionV3, VGG19, and VGG16 models performed optimally on the datasets when weights of 0.5, 0.3, and 0.2, respectively, were assigned to their prediction into the ensemble. Sedik et al. [19] demonstrated that utilizing data augmentation improves detection performance by 11% whenever data are insufficient. They used a deep convolutional generative adversarial network (DCGAN) to generate augmented images which were then used to train deep learning models for COVID-19 detection in CXR. Ismael et al. [5] reviewed many deep learning models for detection in CXR images and showed that fine-tuning recent deep CNNs on COVID-19 datasets achieves excellent detection performance. Their evaluation showed that using a fine-tuned ResNet-50 model as a feature extractor with the SVM classifier yielded optimal performance. In a similar study, Luz et al. [20] fine-tuned EfficientNet model to detect COVID-19 in CXRs. These models are constructed automatically by combining optimal units to achieve the best performance at low cost. Their model can detect COVID-19 pneumonia and can also differentiate it from non-COVID pneumonia. They also evaluated their model on a different dataset to show that the model express generalization. Tackling the problem of insufficient data, Gupta et al. [21] also fine-tuned a number of pre-trained deep CNNs and achieved high detection accuracy. Zhang et al. developed an anomaly detection system where they attempted to spot novel abnormalities as anomalies from CXRs. Their system learned existing pulmonary disorders and their confidence scoring mechanism reports anomaly when an unseen pattern is perceived.

Recent studies have also investigated severity estimation and disease progression and prognosis prediction frameworks to show that such systems can effectively serve in automatic patient triaging, particularly in emergency situations. Alvarez et al. [22] proposed a framework to predict mortality risk using deep learning on 48 different clinical attributes. Their method achieved an accuracy of 95% with the deep learning model. Signoroni et al. [23] proposed a multi-purpose network to segment and align lung regions, detect COVID-19 pneumonia, and output severity scores. These scores were computed on the basis of lung involvement determined by dividing the lungs into six non-overlapping regions. The severity scores obtained from expert radiologists were then used to train a regression head on a large dataset. The model was able to achieve a mean absolute error (MAE) of 1.8. In a similar work by Cohen et al. [24], a version of the DenseNet [25] model was trained on 18 commonly found chest pathologies from several publicly available datasets [26]. Spread of infection and opacity scores provided by three expert radiologists were then used to train a linear regression model. Amer et al. [27] proposed a framework where pneumonia detection and localization models for CXRs were simultaneously trained. The ratio of infected regions as identified via the localization maps to the overall lung region was used to estimate infection severity. In another study, Blain et al. [28] used a similar approach where a U-Net model was trained to segment lungs and a DenseNet121 [25] model to detect lung abnormalities such as interstitial and alveolar opacity. Information from both models was then used to estimate disease severity in CXRs. A model presented in [29] predicted hospital admission for COVID-19 in the general population using demographic, clinical, and laboratory variables. They developed and validated their model using data from two large cohorts in Spain and Italy. They reported good discrimination (area under the receiver operating characteristic curve [AUC] > 0.8) and calibration (slope close to 1) in both cohorts. In another study, Gentilotti et al. [30] assessed COVID-19 progression on day 5 from symptoms onset using clinical features and laboratory parameters. They analyzed data from 1021 patients admitted to a hospital in France with confirmed COVID-19 infection. They identified male sex, age > 65 years, dyspnea, cardiovascular disease, and at least three abnormal laboratory parameters as predictors of COVID-19 progression. They reported moderate discrimination (AUC = 0.74) but poor calibration (Hosmer–Lemeshow test *p* < 0.001) for their model.

Tremendous success has been achieved using deep learning techniques where end-to-end models are trained on large datasets. In addition to those frameworks, content-based image retrieval (CBIR) provides an alternative method for medical image analysis, particularly when data are insufficient to effectively train novel models. In CBIR, a query image is provided against a large dataset and a small set of relevant images/cases are retrieved. In medical imaging, CBIR has been extensively investigated for its potential applications in many areas, such as content-based access to pathology images which can be used by pathologists to reach diagnosis. During the pandemic situation, searching of relevant information like case reports, along with visually similar radiographs, can prove to be highly beneficial for both physician and radiologist. Innumerous studies exist on the retrieval of visually and semantically relevant CXRs [1,3,4,31,32,33]. Similar approaches have also been developed for retrieval of images along with associated clinical records in COVID-19 patients [34,35,36,37,38]. Though case retrieval has been extensively studied as computer aided diagnosis methods, the potential of relevant case analysis for progression and prognosis prediction in COVID-19 patients requires extensive investigation.

## 3. Materials and Methods

**Figure 1:**
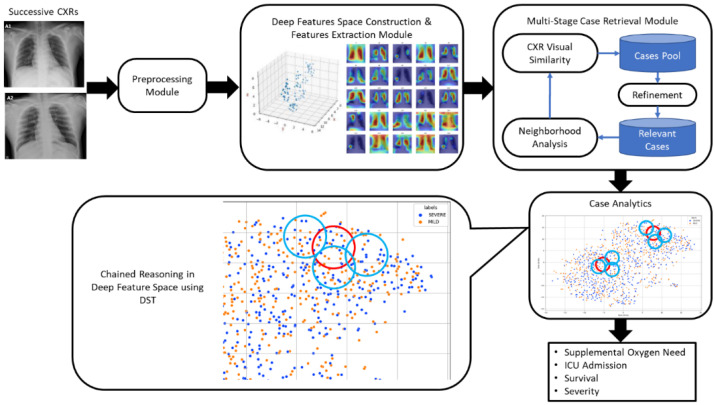
Proposed Framework.

This section presents the proposed framework for progression and prognosis prediction for COVID-19 patients using the relevant case retrieval method assisted by deep features of chest radiographs and a reasoning process. The framework provided in Figure 1 shows the various modules involved, including preprocessing, COVID-specific deep feature space construction and features extraction, multi-stage case retrieval, and a reasoning module for prognosis prediction.

The main goal of this framework is to predict the likelihood of disease progression and clinical outcomes for COVID-19 patients based on their chest radiographs and other clinical data. This can help clinicians make timely and informed decisions about patient management and treatment strategies. To achieve this goal, the framework uses a relevant case retrieval method that leverages deep features of chest radiographs to find similar cases from a historical database of COVID-19 patients with known outcomes [39]. The retrieved cases are then used as inputs for a reasoning module that applies Dempster–Shafer theory to estimate the prognosis of the current patient. The framework also incorporates a COVID-specific deep feature space construction module that adapts a pre-trained convolutional neural network (CNN) to extract discriminative features from chest radiographs that capture the severity and extent of lung involvement by COVID-19 patient. The preprocessing module performs image enhancement, resizing, and normalization on the chest radiographs before feeding them to the feature extraction module. Further details of each module are provided in the subsequent sections.

### 3.1. Study Population

Two datasets have been used in this study. The first one is the AIforCOVID dataset [40] which includes data collected from 820 patients (1105 scans) gathered from six Italian hospitals during the first COVID-19 emergency in spring 2020. The dataset includes chest X-rays, clinical attributes, and outcomes. This dataset was collected to assess the potential of artificial intelligence to predict the prognosis of such patients, distinguishing between severe and mild cases. In addition to that, other attributes provided in the dataset are patient age, supplemental oxygen need, survival, prognosis (Mild, Severe), and respiratory failure, etc. The second dataset is the COVID chest X-ray dataset [41] which contains 930 scans from 472 patients. Attributes in this dataset include age, supplemental oxygen need, survival, ICU, intubation, and severity. The common attributes from both datasets have been used in this study.

### 3.2. Analysis of Chest Radiographs and Associated Records

Patients’ electronic health records (EHR) consist of biodata, clinical findings including lab tests, radiographic scans, and associated reports. Records for a particular patient gathered over time can reveal useful insights into the patient’s health and its deterioration and improvement during a particular disease. In the case of COVID-19, these records can prove to be extremely useful during diagnosis, treatment, and prognosis prediction based on past records of similar patients. This relevance of records can be determined based on many factors including age, gender, lab test reports, radiograph similarity, and many others. An effective method to determine record similarity in COVID-19 patients is crucial, particularly when used in case-based reasoning. The most challenging and critical component of the record is a radiograph which contains useful information about the type of infection, the extent of spread, and the type of abnormalities introduced by the infection. This visual information in CXRs being highly unstructured requires highly discriminative features which is often carried out using deep learning.

### 3.3. CXR Preprocessing

**Figure 2:**
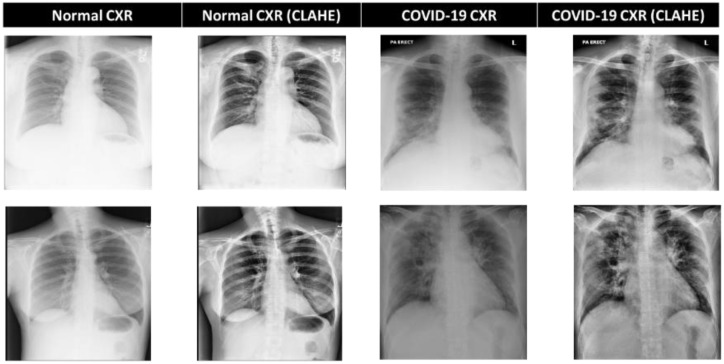
Sample scans before and after image enhancement.

In a typical end-to-end learning framework, input images are forwarded to the network without any significant preprocessing. This approach is fine for natural images which usually do not have any issues related to exposure or contrast. However, in our case (i.e., chest X-rays), we believe that as the physicians can benefit from an appropriately processed CXR during diagnosis, the features extraction in deep learning models can also be improved [42]. In this regard, we evaluated various sets of preprocessing approaches for deep learning of CXRs and found that the contrast limited adaptive histogram equalization (CLAHE) method can prominently highlight the anatomical structures and other regions of interest in CXRs for easy visualization and interpretation. It effectively eliminates issues arising from uneven illumination which makes it difficult to observe darker and overly bright regions. It helps highlight important characteristics of CXRs such as consolidation, improves infiltrates appearance due to adaptive/local contrast adjustment, and enhances minute details which can be highly beneficial for the early detection of an infection. Figure 2 presents CXRs from both normal and COVID-19 patients. The unprocessed CXRs appear foggy, have uneven illumination, and the details which distinguish a normal CXR from the one with an infection are not prominently visible. Enhancements obtained via CLAHE depicted in the second and fourth columns reveal the differences in both normal and COVID-19 infected patients. Elaboration of critical regions in CXRs will allow deep learning models to effectively model subtle differences.

### 3.4. COVID-Specific Deep Feature Space Construction and Features Extraction

Features extraction is the most crucial part of any visual recognition system. CNNs have proven to be excellent features extractors which serve as the backbone for many visual recognition tasks involving classification, object detection and localization, and image segmentation. The features extraction module in our framework consists of a fine-tuned DenseNet121 model which is pretrained on CheXpert Dataset. This pre-trained model has shown comparable performance to an expert radiologist in identifying 18 chest pathologies in CXRs. In our case, we fine-tuned this model on a COVID-19 dataset which consists of normal and COVID infected radiographs. As a result of the fine-tuning, the features extraction pipeline was optimized to detect anomalies in CXRs. This model was then used to construct a feature subspace for representing COVID-infected patients.

**Figure 3:**
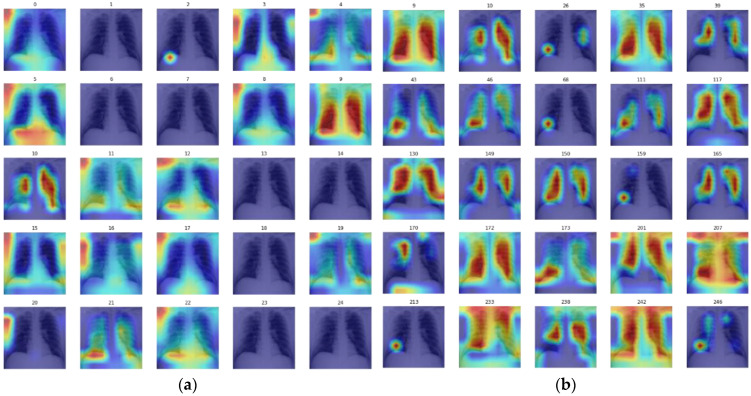
(**a**) First 25 feature maps from the last convolutional layer, (**b**) First 25 selected feature maps overlayed on the input image.

Typically, the activations of the final layer are used as features to represent images. We argue that the activations maps from convolution layers can serve as better descriptors for CXRs than the global features obtained from fully connected layers, particularly when spatial information of neuronal activations are useful in decision making. To obtain COVID-19-specific features from the model, we analyzed individual layers of the model to identify the most descriptive sets of features from the deeper convolutional layers in terms of their response to COVID specific abnormalities. Visualizations of neuronal activations on CXRs were observed with the assistance of a radiologist and optimal features were selected for use in the subsequent modules. Particularly, features that correspond to the infected areas of the CXR were selected and the rest were ignored. This helped in dimensionality reduction as well as elimination of insignificant and trivial features. Samples of the selected convolutional features have been provided as overlapped regions on input CXR in Figure 3b. Algorithm 1 presents the procedure for feature space construction, whereas Algorithm 2 discusses the features extraction process.
**Algorithm 1: COVID-Specific Deep Feature Space Construction**1:**Input:** Image Set (T) consisting of2:I.Chest X-Ray images from COVID Patients3:II.ROI coordinates of the affected regions4:**Output:**5:Feature Subspace dimensions F_S_ (Indices of the selected neurons)6:**Preparation:**7:I.Initialize the DenseNet-121 model.8:II.Initialize the neuronal activation index (NAI) having size length(T) x FN to zeros.9:**Steps:**10:I.**for each** image t in T11: a.Forward propagating all the ROI labeled CXRs through the DenseNet-121 model.12:     
$F_{X}=f_{DenseNet121}(X)$
13: b.Extract h x w x F_N_ activation maps from the final convolutional layer14: c.Find location of the max activation and select the neuron if the location of max activation is contained within the ROI.15: d.Mark the index of the selected neuron in the NAI with one.16:**end for**17:II.Feature subspace is constructed using the marked neurons as F_S_18:III.Return NAI & F_S_


**Algorithm 2: Features Extraction and Representation**
1:**Input:** CXR2:**Output:** Feature Vector F_x_3:
**Steps:**
4:
I.Forward propagate the input CXR through the DenseNet-121 fine-tuned model
5:
II.Obtain the 1024 × 7 × 7 tensor from the last convolutional layer.
6:
III.Extract the selected maps using the indices of the chosen maps.
7:
IV.Normalize activations xn and binarize xn using threshold τ to obtain bn
8:     
$x_{n}=\frac{x-\mu }{max(x)-min(x)}$
9:     
$b_{n}=\left\{\begin{array}{l} 1,\;x_{n}>\tau \\ 0,\;Otherwise \end{array}\right\}$
10:
V.Take a global sum of each individual map to obtain the feature vector F_x_
11:
VI.Return F_V_


**Figure 4:**
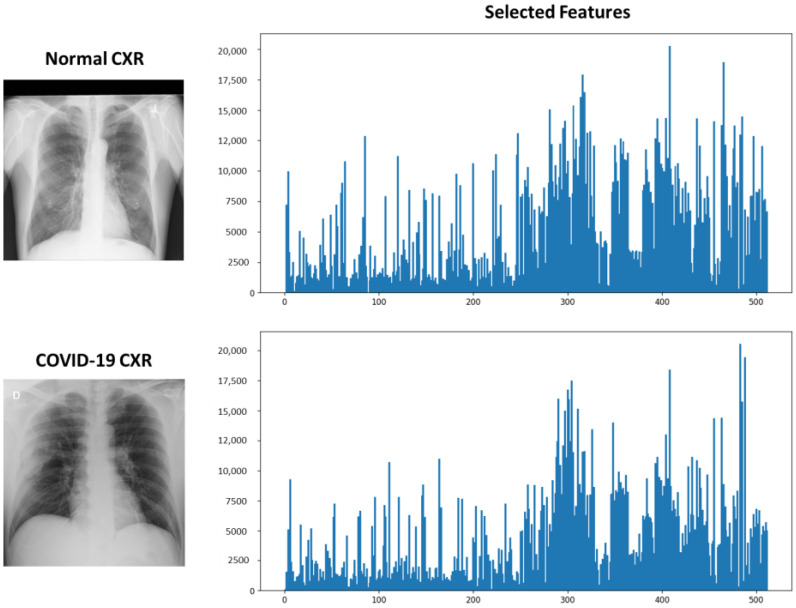
Sample CXRs and their corresponding feature vectors.

The activations maps are then transformed into a feature vector using global average pooling where each feature map is averaged into a single value. This value corresponds to the strength of the feature for the input CXR. Higher values represent a strong presence, whereas lower values mean weaker presence of that particular feature. Since we are only selecting those features which are sensitive to infected regions, their presence and strength can be effectively determined by the feature vector. Sample feature vectors for both normal and COVID-19 CXRs are provided in Figure 4. The features of COVID-19 CXRs are different than the features of a normal one. Furthermore, the similarity in features corresponds to the presence of similar pathologies in the CXRs since the feature space is constructed by only considering infection sensitive neurons.

The activations obtained from the selected maps are then accumulated into a combined map to indicate the extent of the spread of infection. This information is then used by the retrieval module to determine similarity, and the reasoning module to determine progression and severity as discussed in the subsequent section.

### 3.5. Case Retrieval Using CXR Similarity

Each patient record may consist of several vital clinical variables along with results of lab tests conducted from time to time and the associated radiographic scans. These pieces of information are crucial but are low-dimensional and cannot be effectively embedded into a feature space. Therefore, in this study, we treat them as variables associated with each CXR representing the state of patient at a particular time. This variable association enriches the embedding and allows for accurate estimation of semantic relevance during case retrieval.

**Figure 5:**
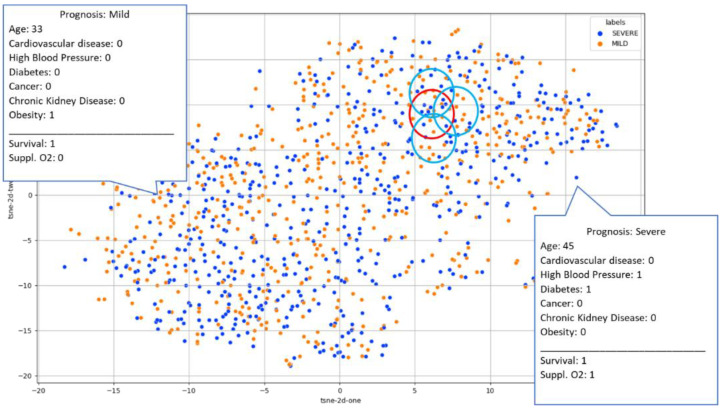
Two-dimensional tSNE embedding of the 512-D feature space along with neighborhoods considered during relevant case retrieval. The red circle represents immediate neighbors whereas the blue circles indicate second stage neighbors. Each point represents a CXR embedding into the feature space along with the associated variables.

The retrieval module determines case similarity based on several parameters, including visual similarity determined by the visual features, and the associated attributes such as age and comorbidities. The most relevant cases are retrieved by examining the neighborhood within the deep feature space by considering the Euclidean distance between the visual features as well as the similarity in age and comorbidities. Firstly, a pool of potentially relevant CXRs is retrieved, then neighbors of each relevant case are further analyzed during the second stage to retrieve more potentially relevant cases. Figure 5 illustrates the two-stage retrieval process where the red circle indicates the neighborhood considered during the first stage and the blue circles indicate the neighborhood considered during the second stage of the retrieval process. We chose this approach after analyzing the t-stochastic neighbor embeddings (tSNE) [43] of the feature space and found that in most cases, the mild and severe cases were mixed. In such a situation, instead of selecting a single larger neighborhood to extract relevant cases, we decided to select multiple small neighborhoods. In the case of two or more successive radiographs, the pool of cases retrieved for each CXR is analyzed to determine progression and prognosis. Table 1 presents the various comorbidities along with their role in disease progression and prognosis that have been considered in this study. Cardiovascular disease, high blood pressure, diabetes, and obesity are comorbidities that increase the risk of severe illness in COVID-19 patients [44,45].

### 3.6. Case Analytics via Deep Feature Space Reasoning

Once the relevant cases are retrieved, the pool of cases (CXRs along with the health records) is analyzed to predict the prognosis of the current patient. The health record of past patients contains details such as age, comorbidities, progression, and survival details. These characteristics are utilized by the reasoning module to determine progression/prognosis and survival of the patient as outlined in Algorithm 3. The retrieved cases are ranked based on their overall similarity with the current patient in terms of visual features, age, and comorbidities. The most similar case is the one where visual as well as health record similarity is maximum among the pool of cases. Each case is weighed based on their ranks. The lower rank cases are assigned higher weights, whereas the lower rank cases carry smaller weights. Each of these weights is then used to compute the effect of that record on the prediction of the current patient.

Dempster–Shafer theory of evidence [46] is used to combine all the evidence present in the form of relevant cases and compute probabilities for the current patient’s survival, the need for supplemental oxygen, and prognosis as either mild or severe. The purpose of decision combination is to summarize and simplify information rationally obtained from independent and multiple sources (which in our case correspond to the neighboring relevant cases). The combined probability represents the impact of the combined evidence. The combination rule determines the joint mass from the aggregation of two probability assignments using the following equation:

(1) $$m1⊗m2(z)=\frac{\sum_{x,y\subseteq 2^{\theta },x\cap y=z}m1(x).m2(y)}{1-\sum_{x,y\subseteq 2^{\theta },x\cap y=\emptyset }m1(x).m2(y)}$$

where m1 and m2 are the rank-weighted probabilities obtained from the pool of retrieved cases. All incoming scores are combined using (1) and the probabilities for patient survival, O_2_ need, ICU admission, and severity are computed. For a single CXR, the pool of cases contributes towards computation of output probabilities. In the case of multiple CXRs, the incoming pools of cases are used to compute the outputs. Increase or decrease in the confidence of the outputs can be interpreted as improvement or deterioration.
**Algorithm 3: Deep Feature Space Reasoning for Prediction**1:**Input**:2:Chest X-ray features (512-D) denoted by F_X_ and clinical variables including comorbidities (Cardiovascular disease, High Blood Pressure, Diabetes, Cancer, Chronic Kidney Disease, and Obesity) denoted by C.3:**Output:**4:Progression variables (need for supplemental oxygen, ICU admission, survival, and prognosis) denoted by P.5:**Steps:**6:Deep Feature Space Neighborhood Analysis:7: a.Closest neighbors of the input CXR are collected into the pool.8: b.The variables associated with each case are considered to determine relevance and the less relevant cases are ignored. To achieve this, we computed the Euclidean distance between the input CXR and the neighboring CXRs within the 512-D feature space.9: c.Visual similarity between the input CXR and the target CXR is determined by comparing their Euclidean distance with a threshold T_1_.10: d.Further relevance is determined after considering similarities in terms of comorbidities and age.11:2.Evidence collection:12: a.Euclidean distance D_E_ between the input CXR visual features F_X_ and the target CXR F_Y_, age group A, and common comorbidities C (i.e., similar health status) are considered as evidence for further reasoning. The evidence using visual similarity V_E_ is computed as:13:  
$V_{E}=\left\{\begin{array}{l} 1-D_{E},\;D_{E}\leq T_{1}\\ 0,\;Otherwise \end{array}\right\}$
14:  where $D_{E}=\frac{\sqrt{\sum_{i=1}^{n}\left|x_{i}-y_{i}\right|}}{T_{1}}$ is the normalized distance between X and Y.15: b.Evidence for comorbidities C_E_ is computed as:16:  $C_{E}=\frac{M-\sum_{i,j=1}^{M}\left|c_{i}-c_{j}\right|}{M}$ where M is the number of comorbidities considered.17: c.Evidence for age is computed as:18:  $A_{E}=\left\{\begin{array}{l} 1-\frac{A_{X}-A_{Y}}{R},\;\frac{A_{X}-A_{Y}}{R}\leq 1\\ 0,\;Otherwise \end{array}\right\}$
19: where R is the maximum age difference in an age group.20: d.Evidence of survival S, ICU admission I, prognosis P, and the need for supplemental oxygen O21:  $S_{x}=\frac{\sum_{y=1}^{N}S_{y}}{N}$, $I_{x}=\frac{\sum_{y=1}^{N}I_{y}}{N}$, $P_{x}=\frac{\sum_{y=1}^{N}P_{y}}{N}$, $O_{x}=\frac{\sum_{y=1}^{N}O_{y}}{N}$22:  where S_x_ is the probability of survival of patient x, I_x_ represents ICU admission, P_x_ determines prognosis (mild vs. severe), and O_x_ identifies as the need for supplemental oxygen. N represents the number of relevant cases considered.23:3.Evidence combination:24: a.Assign belief masses (i.e., weights) to each chest X-ray feature, clinical variable, and progression variable based on their relevance to the prediction problem.25: b.Use Dempster’s rule of combination to combine the evidence from multiple sources. Use Equation (1) iteratively to combine all the evidence and obtain the probabilities for progression (I, S, P, and O) and prognosis.26:  In Equation (1), m1 and m2 are the belief values (probabilities) of relevant cases for the parameters x and y. For instance, if survival is to be predicted, then the survival of all relevant cases will be combined via the above function to obtain a survival prediction for the current patient.27:4.Return progression and prognosis predictions.

### 3.7. Progression and Prognosis Prediction

Retrieved cases serve as evidence gathered by the retrieval module and are analyzed to estimate how likely a current patient is to develop complications or recover from their condition. For any patient under study, multiple relevant cases may be retrieved, each with a different degree of similarity with the current patient. Based on the rank, the evidence (relevant cases) is weighted and each output parameter including the need for supplementary O_2_, ICU admission, survival, and prognosis (mild or severe) are computed using the method outlined in Algorithm 3. The output parameters obtained after analyzing all the evidence for all patients, an effective triaging mechanism could easily be put in place during emergency situations. The predictions help us determine whether they need extra oxygen or intensive care unit (ICU) admission, whether they will survive or not, and whether their condition is mild or severe. These parameters can help us decide how to prioritize and allocate resources (like oxygen and ICU beds) for different patients in emergency situations. Our method is useful because it can work even when we have only a few cases in our database. The nomenclature used in the proposed framework is provided in Table 2.

## 4. Experimental Results and Analysis

### 4.1. Case Retrieval Performance

**Figure 6:**
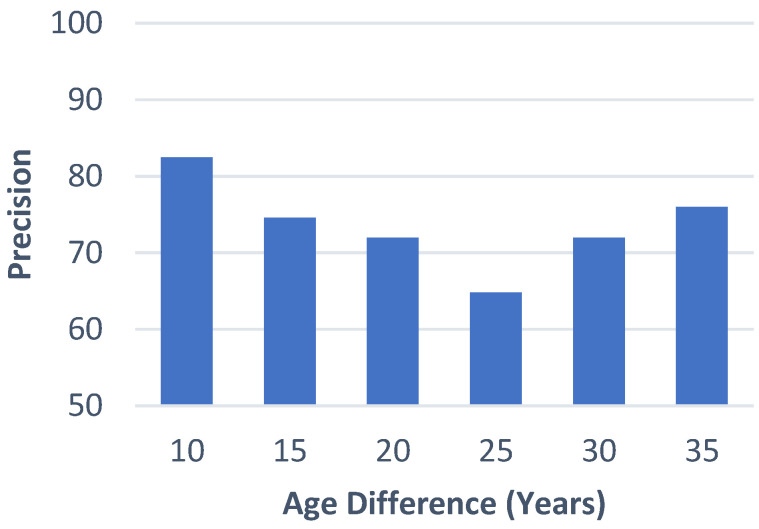
Effect of age difference in case retrieval on severity prediction performance.

The retrieval of relevant cases is highly crucial for the accurate operation of the proposed framework. The case matching and retrieval module is thus a key component of our method. To achieve optimal performance, we evaluated the method by modifying various parameters such as age range and comorbidities. This has already been established, that age and comorbidities are highly crucial when diagnosing COVID-19 or predicting its progression. These are vital to consider for computing case similarities. Figure 6 show the varying age differences that were considered for case retrieval. The optimal age range to consider was found to be around 10 years.

**Figure 7:**
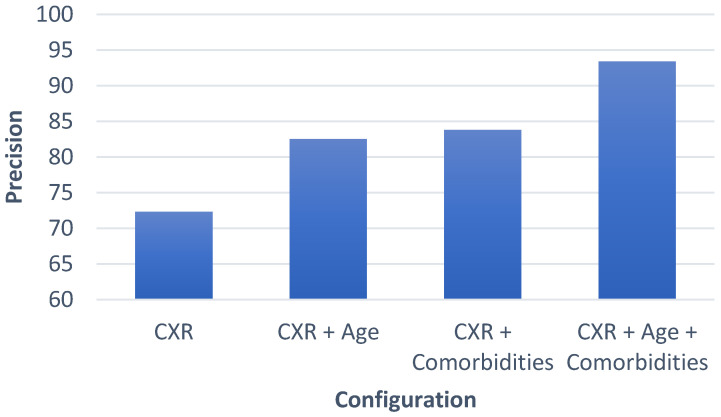
Effect of various parameters in case retrieval on severity prediction performance.

Another factor to consider for case similarity was comorbidities. In this regard, we evaluated various configurations (as shown in Figure 7) to determine the optimal scheme for case matching. CXR similarity alone achieved 72% precision, which increased to 83% when age was included with it. Age is a significant factor in predicting COVID-19 progression and prognosis as older individuals are more susceptible to severe illness and are at a higher risk of hospitalization and death. In addition, older individuals are more likely to have underlying medical conditions such as cardiovascular disease, high blood pressure, and diabetes, which can worsen COVID-19 outcomes. Combining CXR and comorbidities resulted in 84% precision and the best results were obtained when CXR, age, and comorbidities were used to retrieve relevant cases. This is because these factors can provide important information about a patient’s overall health status and their likelihood of developing severe illness.

**Figure 8:**
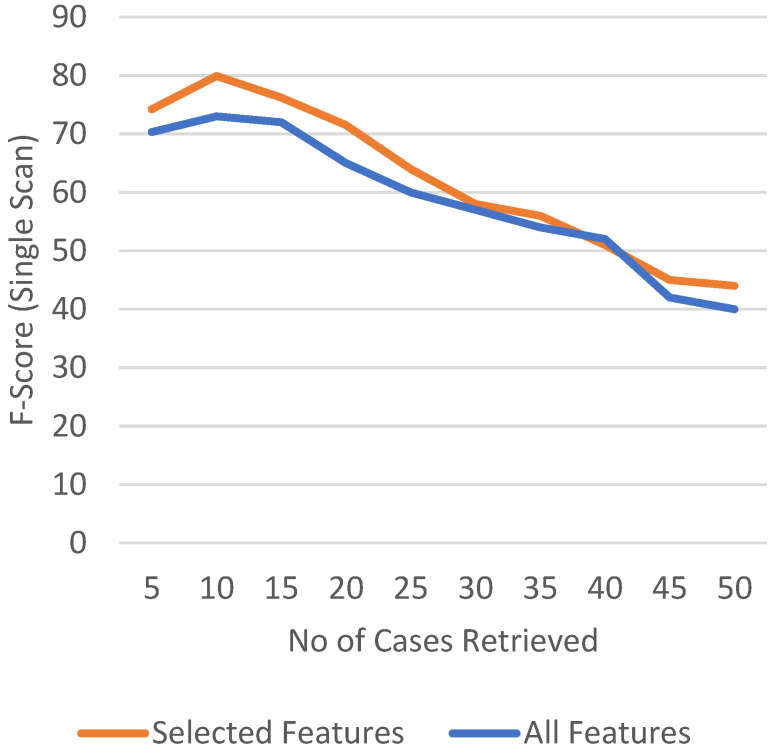
Comparison of selected features for single scan.

**Figure 9:**
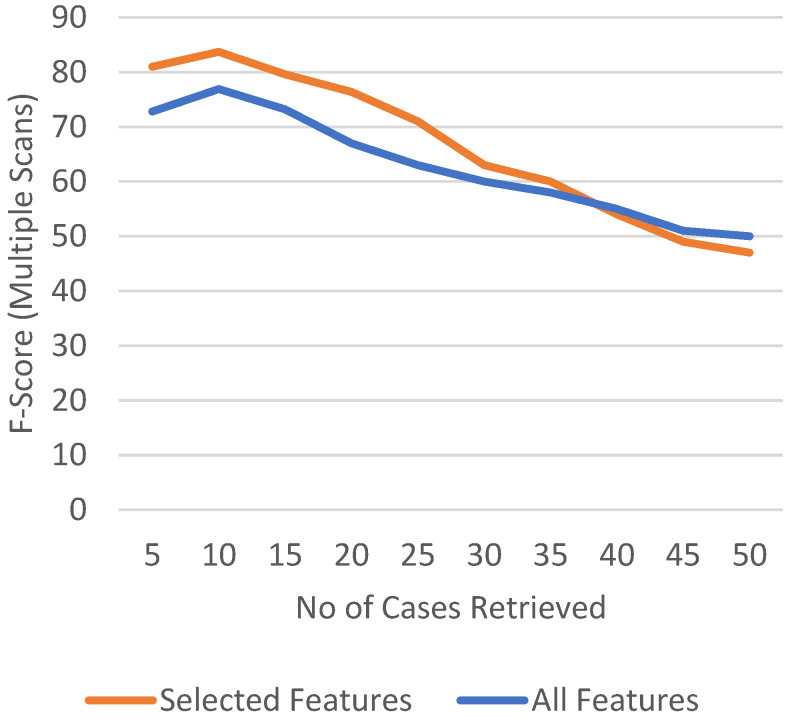
Comparison of selected features for multiple scans.

CXR similarity is very challenging, particularly when fine-grained differences and similarities need to be detected. For this purpose, we proposed a method to represent CXRs with COVID-19-specific features. Results depicted in Figure 8 reveal that the use of selected features instead of all features from a deep convolutional layer yields better performance when a single scan is available. Figure 9 shows the performance enhancement when multiple scans are available for predicting severity.

### 4.2. Retrieved Cases (Images with Clinical Records)

**Figure 10:**
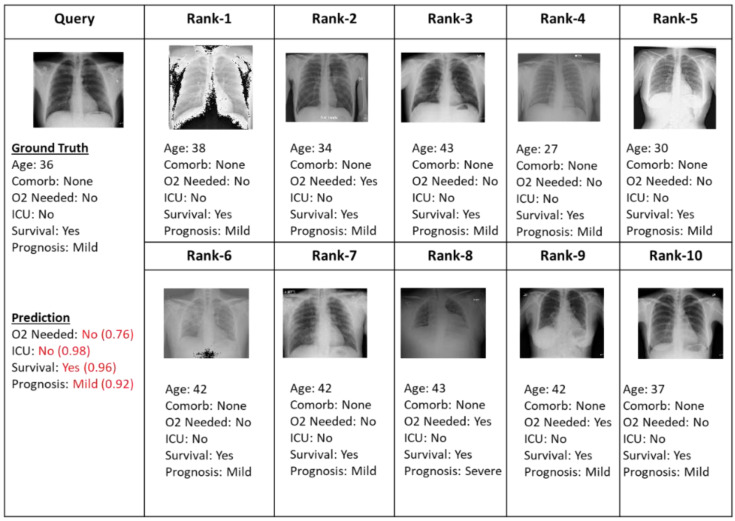
Top 10 relevant cases retrieved for the query case (Patient age = 36 years).

**Figure 11:**
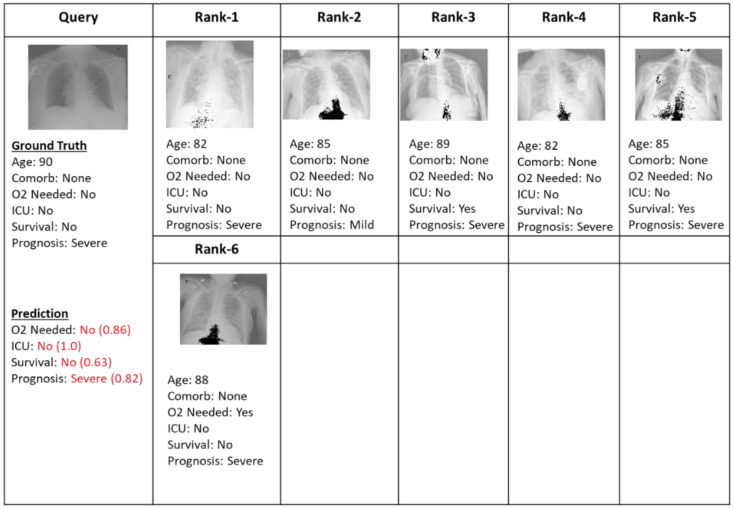
Top 7 relevant cases retrieved for the query case (Patient age = 90 years).

In this experiment, we used the COVID-19-specific features to represent CXRs to retrieve a pool of cases from the dataset. In the second phase, age and comorbidities were used to shortlist the most relevant cases. The diagnosis reports and patient history was then analyzed to determine patient survival, prognosis, ICU admission probability, and the need for supplemental oxygen. Top k retrieved cases for two patients are shown in Figure 10 and Figure 11, where the first patient is 36 years old and the second one is 90 years old. In Figure 10, ten cases were retrieved from the dataset along with their findings, and history. The details (ground truth and predictions) for the query patient are also presented for comparison. It can be seen that the top-ranked patients have similar readings regarding their survival, ICU admission, and oxygen need. The majority of the patient’s prognosis is mild, whereas just one patient at rank 8 had severe infection. Taking into consideration the history of similar patients, it can be witnessed that the correct predictions for the current patient can be performed with confidence. In Figure 11, six cases were retrieved for the query from the same age group. None of the patients had any comorbidities, which makes them similar to the input patient. The majority of the patients (5/6) did not need supplemental oxygen. None of the patients were admitted to the ICU. Four of the six patients did not survive the infection and the prognosis of 5 out of 6 patients was severe. Based on these reports, the progression and prognosis for the query patient can be predicted accurately. In these experiments, it was observed that the progression and prognosis of younger patients can be predicted more consistently than the older patients.

### 4.3. Progression/Prognosis Prediction

The prediction of severity and survival of COVID-19 patients become highly crucial in emergency situations. It becomes desirable to have an effective automated triaging system. The proposed method was evaluated on the test datasets and the results are reported in Table 3 and Table 4. Table 3 lists the performance of the proposed method with a single scan. For supplemental oxygen, a 0.822 F-score was obtained. For ICU admission, survival, and severity, scores of 0.809, 0.787, and 0.780 were achieved, respectively. Similarly, in Table 4, performance with multiple scans is reported where significant performance improvement is noticed. The results indicate that our method is capable of predicting progression/prognosis and survival with a high degree of precision. The reason for the superior performance with multiple scans is the availability of more evidence for ensuring high confidence predictions.

### 4.4. Comparison with Similar Methods

Several similar works exist in the literature that utilized visual modalities like CXRs and CT, along with clinical variables or comorbidities in COVID-19 patients as presented in Table 5. Jiao et al. [47] used CXRs as input to an EfficientNet deep neural network and clinical data to train models and predict the binary outcome of disease severity (i.e., critical or non-critical). The deep-learning features extracted from the model and clinical data were used to build time-to-event models to predict the risk of disease progression. Their method was able to achieve a 0.830 F-score on the test set. In a similar work by Schalekamp et al. [48], a risk model to predict critical illness (i.e., death and/or intensive care unit admission with invasive ventilation) was developed, using multivariable logistic regression, including clinical, chest radiographic, and laboratory findings. Their method was able to achieve a 0.826 F-score on the test set. Gong et al. [8] and Feng et al. [11] proposed frameworks to predict the progression and prognosis of patients using CT scans along with clinical variables. Though they used a different modality, the approach is similar in terms of considering multi-modal attributes of patients and proved effectiveness of the method. Our method has the distinct advantage that it can work with single as well as multiple scans to retrieve relevant cases from the dataset and then the effective reasoning strategy utilizes the gathered evidence to predict progression and prognosis. Experiments revealed that the proposed method achieved a 0.835 precision with a single scan and 0.881 with multiple scans. Though currently, we have only experimented with two CXRs, the availability of multiple scans will further improve prediction.

### 4.5. Discussion

Chest x-rays have long been a crucial diagnostic tool in the management of respiratory illnesses. In the context of COVID-19, chest X-rays can provide valuable information about the progression of the illness, including the presence of pneumonia and the extent of lung involvement. This information can help healthcare providers to diagnose the illness more accurately and to better understand the potential outcomes for the patient.

When considered along with age and comorbidities, chest X-rays can provide a more comprehensive picture of a patient’s health status and can help to predict the progression and prognosis of the illness more accurately. Age is a significant factor in the progression and prognosis of COVID-19 as older individuals are more susceptible to severe illness and are at a higher risk of hospitalization and death. Comorbidities such as cardiovascular disease, high blood pressure, diabetes, and obesity can also increase the risk of severe illness and can affect the body’s ability to fight off the virus and manage symptoms.

The combination of chest X-rays and information about age and comorbidities can help healthcare providers to make more informed treatment decisions and to provide a more accurate prognosis for patients. For example, in cases where a patient has a high risk of severe illness based on age and comorbidities, a chest X-ray showing extensive lung involvement may prompt a more aggressive treatment approach. Conversely, in cases where a patient has a lower risk of severe illness, a chest X-ray showing only mild lung involvement may indicate a more conservative treatment approach.

## 5. Conclusions and Future Work

This paper presented a method which utilizes visually and semantically similar CXRs of COVID-19 patients to predict disease progression and prognosis. Firstly, a pool of cases was retrieved from the dataset using visual similarity of CXRs. This was achieved with the help of COVID-19-specific features which were chosen using a novel feature selection algorithm in a DenseNet121 model trained on chest pathologies. Only those neurons which reacted actively to COVID-19 affected regions in the CXR were selected. These features were then used to retrieve visually and semantically similar CXRs from the dataset. From this pool of cases, the most relevant cases were chosen based on age group and comorbidities. These relevant cases were then used in a Dempster–Shafer theory of reasoning to predict progression and prognosis. The reasoning module computes case similarity scores and uses them to calculate weighted probabilities for ICU admission, need for supplemental oxygen, prognosis, and survival.

Experiments carried out using the proposed framework reveal that progression and prognosis can be accurately predicted from past cases when they are retrieved using this method. Furthermore, the reasoning process presented can combine evidence from multiple cases as well as multiple radiographs. It was also shown that when multiple scans are available for a patient, the prediction performance of the proposed method is improved.

We intend to improve the proposed method further by building an end-to-end learning and reasoning method. A dynamic reasoning module integrated into the overall learning framework can further improve performance of the proposed method.

## Figures and Tables

**Table 1:** Comorbidities considered in this study in COVID-19 patients.

SNo	Comorbidities	Description
1	Cardiovascular disease	COVID-19 can cause stress on the heart and blood vessels, leading to a higher likelihood of cardiovascular events such as heart attack or stroke.
2	High Blood Pressure	High blood pressure can also worsen COVID-19 outcomes as it increases the risk of severe illness.
3	Diabetes	Diabetes can affect the body’s ability to fight off the virus and manage symptoms, while obesity can increase the risk of hospitalization and respiratory failure.
4	Cancer	Cancer patients, especially those undergoing treatment, have a weakened immune system which can make them more susceptible to severe COVID-19.
5	Chronic Kidney Disease	Chronic Kidney Disease increases the risk of hospitalization, mechanical ventilation, and death in COVID-19 patients as it affects the body’s ability to clear waste and fluid.
6	Obesity	Obesity can put additional strain on the respiratory system, making it harder for the body to fight off the virus and manage symptoms. This can increase the risk of respiratory failure and the need for mechanical ventilation.

**Table 2:** Description of nomenclature used.

Model Parameters	Description	Model Parameters	Description
T	Labeled image set	τ	Threshold value
F_S_	Feature subspace	V_E_	Visual similarity
X	Input CXR	D_E_	Normalized distance between X and Y
Y	Target CXR to be compared for relevance	C_E_	Evidence corresponding to comborbidities
F_x_	Deep features of X	A_g_	Evidence for Age
NAI	Neuronal activation index	S_x_	Probability of survival of patient x
I_x_	ICU admission	P_x_	Prognosis (mild vs. severe)
O_x_	Need for supplemental oxygen		

**Table 3:** Prediction performance of the proposed method using single scan per patient (both datasets).

Prognosis (Single Scan)	Precision	Recall	F-Measure
Supplemental Oxygen	0.85	0.796	0.822
ICU Admission	0.84	0.78	0.809
Survival	0.86	0.725	0.787
Severity	0.79	0.77	0.780
**Overall**	**0.835**	**0.768**	**0.799**

**Table 4:** Prediction performance of the proposed method using multiple scans per patient (both datasets).

Prognosis (Multiple Scans)	Precision	Recall	F-Measure
Supplemental Oxygen	0.86	0.802	0.830
ICU Admission	0.846	0.815	0.830
Survival	0.882	0.79	0.833
Severity	0.936	0.784	0.853
**Overall**	**0.881**	**0.798**	**0.837**

**Table 5:** Comparison of the proposed method with similar existing methods.

Severity Prediction Methods	Precision	Recall	F-Measure
Jiao et al. [47] (CXR + Clinical)	0.853	0.738	0.830
Schalekamp et al. [48] (CXR + Comorbidities)	-	-	0.826
Gong et al. [8] (CT + Age + Comorbidities)	0.702	0.905	0.788
Feng et al. [11] (CT + Clinical)	-	-	0.820
Proposed Method (Single Scan)	0.835	0.768	0.799
Proposed Method (Multiple Scans [2+])	0.881	0.798	0.837

## Data Availability

Data and relevant material will be made available at: https://github.com/jamilahmadicp/COVID-19-Progression accessed on 16 March 2023.
